# Automated GMP Production and Preclinical Evaluation of [^68^Ga]Ga-TEoS-DAZA and [^68^Ga]Ga-TMoS-DAZA

**DOI:** 10.3390/pharmaceutics14122695

**Published:** 2022-12-01

**Authors:** Julia Greiser, Thomas Winkens, Olga Perkas, Christian Kuehnel, Wolfgang Weigand, Martin Freesmeyer

**Affiliations:** 1 Clinic of Nuclear Medicine, University Hospital Jena, Am Klinikum 1, 07747 Jena, Germany; 2 Institute for Inorganic and Analytical Chemistry, Friedrich Schiller University, Humboldtstrasse 8, 07743 Jena, Germany

**Keywords:** Gallium-68, PET, radiopharmacy, liver diagnostics, imaging, N,O ligands, DAZA, in ovo model

## Abstract

[^68^Ga]Ga-TEoS-DAZA and [^68^Ga]Ga-TMoS-DAZA are two novel radiotracers suitable for functional PET liver imaging. Due to their specific liver uptake and biliary excretion, the tracers may be applied for segmental liver function quantification, gall tree imaging and the differential diagnosis of liver nodules. The purpose of this study was to investigate problems that occurred initially during the development of the GMP compliant synthesis procedure and to evaluate the tracers in a preclinical model. After low radiolabeling yields were attributed to precursor instability at high temperatures, an optimized radiolabeling procedure was established. Quality controls were in accordance with Ph. Eur. requirements and gave compliant results, although the method for the determination of the ^68^Ga colloid is partially inhibited due to the presence of a radioactive by-product. The determination of logP revealed [^68^Ga]Ga-TEoS-DAZA (ethoxy bearing) to be more lipophilic than [^68^Ga]Ga-TMoS-DAZA (methoxy bearing). Accordingly, biodistribution studies in an in ovo model showed a higher liver uptake for [^68^Ga]Ga-TEoS-DAZA. In dynamic in ovo PET imaging, rapid tracer accumulation in the liver was observed. Similarly, the activity in the intestines rose steadily within the first hour p.i., indicating biliary excretion. As [^68^Ga]Ga-TEoS-DAZA and [^68^Ga]Ga-TMoS-DAZA can be prepared according to GMP guidelines, transition into the early clinical phase is now possible.

## 1. Introduction

*N*,1,4-Tri(4-alkoxy-2-hydroxybenzyl)-1,4-diazepan-6-amine (if alkoxy = ethoxy: TEoS-DAZA; if alkoxy = methoxy: TMoS-DAZA) are novel chelators that are able to coordinate ^68^Ga(III) ions, thereby forming liver-specific ^68^Ga complexes which are suitable diagnostics for molecular imaging [1]. ^68^Ga, a short-lived (half-life 68 min), positron-emitting radionuclide, is the basis for a great variety of radioactive pharmaceuticals (called “tracers” or radiopharmaceuticals) that are applied in sub-nanomolar amounts in nuclear medical imaging [2,3]. Following tracer injection, its distribution is detected by cameras and turned into three-dimensional images. The visualization and quantification of the tracer distribution and accumulation enables nuclear medical physicians to make accurate diagnoses, because each tracer is designed to suit a specific diagnostic question. Depending on its chemical structure a radiopharmaceutical specifically targets a predefined substrate in the body, e.g., an over-expressed tumor cell receptor or a specific membrane transporter. With the continuous growth of new radiopharmaceuticals, the field of the application of nuclear medical imaging is constantly broadened [4]. There are two different nuclear imaging techniques which are distinguished on the basis of the emitters used: single photon emission tomography, which uses gamma-emitting radionuclides (SPECT), and positron emission tomography (PET), which features the detection of coincidence events caused by two photons of 511 keV that originate in the decay of beta plus emitters. Given that PET combines the acquisition of three-dimensional images of a high spatial resolution with a high temporal resolution of up to three seconds, one of its strengths lies in the possibility to visualize fast dynamic processes [5,6]. Furthermore, it is a quantitative imaging modality by nature [6].

Since liver-related diseases are among the most common causes of death, precise liver diagnostics are crucial [7]. Besides the differential diagnosis of liver nodules and the imaging of the gall tree, an important diagnostic challenge is the exact quantification of functional liver remnants in planned liver resections. While laboratory analyses (LiMAx, indocyanine green ICG clearance) allow one to assess the global liver function, they cannot represent the heterogeneous spatial distribution of liver function across the entire organ, which is made up of eight segments [8]. Therefore, functional liver imaging methods are of immense importance, especially prior to planned liver resections wherein the remaining liver function needs to be assessed as precisely as possible to ensure postoperative survival. Compared to perfusion-based models, function-based imaging methods more accurately represent the actual condition of the liver [8,9]. Although there already exist two imaging methods, each one has a drawback: Hepatobiliary SPECT featuring [^99m^Tc]Tc-imino diacetic acid derivatives produces either three-dimensional, static images which do not represent the dynamic changes of tracer distribution or two-dimensional, temporally resolved images [10]. Either way, with SPECT, one dimension is always missing, whereas functional liver PET imaging combines three-dimensional images with a high temporal resolution. Another alternative is hepatobiliary magnetic resonance imaging (MRI) using the liver-specific Gd-based contrast agent (CA) Gd-EOB-DTPA (Primovist^®,^ Bayer Vital GmbH, Leverkusen, Germany). While MRI provides highly resolved three-dimensional images, signal quantification remains difficult and is still not established for liver function quantification. Likewise, the temporal resolution is limited. Furthermore, Gd-based CAs are described to be associated with side effects and have recently been shown to deposit Gd in the body [11]. Last but not least, renal insufficiency, claustrophobia or metal implants are contraindications that impede the limitless use of MRI procedures [12,13].

So far, no PET tracer for functional liver imaging has been established and approved for clinical routines. Detailed reviews on several strategies to create liver-specific substrates, also based on PET nuclides other than ^68^Ga, such as ^18^F, ^11^C or, in rare cases, ^64^Cu, have been presented previously [14,15]. In short, one group of promising substrates is based on bile acid derivatives labeled with ^18^F or ^11^C [16,17,18,19,20]. Other attempts have focused on ^18^F-labeled 2-deoxy-D-galactose, a radiotracer exhibiting potential as a liver function agent [21,22]. However, the radionuclide ^11^C has a rather short half-life of ca. 20 min, which substantially limits the time frame for production, application and imaging, especially where late acquisitions are necessary. While ^18^F (half-life 110 min) is the most commonly used PET nuclide, its application requires sufficient proximity to a cyclotron. In contrast, ^68^Ga is produced from ^68^Ga/^68^Ge generators, enabling the location-independent synthesis of ^68^Ga radiopharmaceuticals. Few PET tracers for functional liver imaging based on ^68^Ga have been reported [14]. Among them, ^68^Ga tracers based on galactosylated and lactosylated albumins show hepatic uptake but poor biliary excretion [23,24], similar to ^68^Ga tracers targeting the liver reticuloendothelial system [25,26].

TEoS-DAZA and TMoS-DAZA (also known as TEOHB-DAZA and TMOHB-DAZA in earlier publications) are hydroxybenzyl-bearing azacycles that are formed by the amine-carbonyl condensation, and subsequent reduction, of 1,4-diazepan-6-amine (DAZA) with three moieties of the respective alkoxysalicyl aldehyde [1]. Both chelators form ^68^Ga complexes that are stable in vivo. In a proof of principle study, we could show that these radiotracers accumulated in the liver within minutes after i.v. injection and were subsequently excreted via the gall bladder into the duodenum [1]. Preclinical data and a first compassionate use suggest that the tracers are suitable for functional liver imaging with PET [1,27]. Due to the minimal amounts of the tracer necessary to produce a signal, there are no contraindications for a nuclear medical imaging procedure (except for pregnancy). Therefore, it was possible to diagnose biliary stent integrity in a case of the compassionate use of [^68^Ga]Ga-TMoS-DAZA in a patient bearing a Cochlear implant, where MRI was not applicable [28]. To enable tracer application in the clinical practice within the regular framework of the German Medicines Law, a GMP-compliant radiosynthesis of the radiotracers was needed. We describe a fully automated radiolabeling procedure for [^68^Ga]Ga-TEoS-DAZA and [^68^Ga]Ga-TMoS-DAZA and additionally present a preclinical evaluation in an in ovo model.

In ovo PET imaging using large bird embryos is a very novel preclinical model that exhibits some advantages over standard animal testing [29]. Firstly, preclinical imaging is usually performed using mice or rats and therefore requires dedicated small animal imaging modalities. Since some bird embryos, particularly of the ostrich (*struthio camelus*), are much larger than those of mice or rats, preclinical imaging can be performed on routine clinical PET scanners used in humans. Secondly, the size of the bird embryo is advantageous regarding better anatomical resolution. Particularly in the case of ^68^Ga radiopharmaceuticals, where small animal imaging resolution is often inhibited by the comparably high positron range [30], the size of the ostrich embryo may compensate for this partial loss in resolution. Thirdly, the purchase and artificial breeding of bird embryos is by far more affordable and easy compared to the keeping of mice or rats, since only an incubator and some standard equipment, such as scales and a candling lamp, are required. In recent years, we have therefore established the in ovo model using large bird embryos, based on the well-established chicken embryo model [29,31,32]. It has been previously shown that many results gained from the chicken embryo model are transferable to in vivo results gained from mammals [33]. There is reason to believe that this also applies to the ostrich embryo model.

## 2. Materials and Methods

### 2.1. Identification of Decomposition Products of TEoS-DAZA

TEoS-DAZA (10 mg) was dissolved in ethanol (1 mL) and hydrochloric acid (1.0 M, 250 µL). HEPES buffer solution (1.5 M, 1 mL, pH 4.5) was added, and the solution was heated for 15 min at 100 °C. HPLC analysis (as described in 2.4) verified the decomposition of TEoS-DAZA to DEoS-DAZA as the main product. The raw mixture was dried under reduced pressure, resuspended in water and loaded onto a column (C18-silica gel). Column chromatography was performed with a gradient from 95.0% A to 0.0% A, using fractions of 25 mL and a respective stepwise reduction of the percentage of A by 10% (A: water/trifluoroacetic acid (99.9/0.1 *v*/*v*), B: acetonitrile/trifluoroacetic acid (99.9/0.1 *v*/*v*)). The eluted fractions (10 mL per fraction) were collected. DeoS-DAZA was contained in elution fraction 21, while EoS-DAZA was contained in fraction 5. Both samples were analyzed via mass spectrometry (MS, electrospray ionization). Mass spectra were recorded with a LTQ Orbitrap mass spectrometer (Thermo Fisher Scientific GmbH, Bremen, Germany).

### 2.2. Radiolabeling of TEoS-DAZA and TMoS-DAZA

For all radiolabeling procedures, ^68^Ga eluate was produced from a ^68^Ge/^68^Ga generator (Eckert & Ziegler AG, Berlin, Germany) using hydrochloric acid (0.1 M) for generator elution (5 mL per elution). TEoS-DAZA was provided in GMP grade by Inflamed pharma GmbH (Jena, Germany). TMoS-DAZA was synthesized in-house according to the published procedure [1]. For the preparation of a TEoS-DAZA or TMoS-DAZA stock solution and the buffer solutions, water of ultrapure grade (ULTREX^TM^, J. T. Baker^TM^, Avantor™ Performance Materials, Center Valley, PA, USA) was used. For radiolabeling, an aliquot of the precursor (120 µg) was freshly diluted in a mixture of ethanol (100 µL) and hydrochloric acid (20 µL, 1.0 M).

GMP production of [^68^Ga]Ga-TEoS-DAZA and [^68^Ga]Ga-TMoS-DAZA was performed on a GRP synthesizer from Scintomics, using single-use, sterilized cassettes for ^68^Ga-peptide labeling along with the provided reagent kits (Scintomics GRP^®^ SC-01-H hardware kit) from ABX GmbH (Radeberg, Germany). The standard ^68^Ga radiolabeling program provided for the synthesizer was modified as follows:

- The initial automated SPE cartridge conditioning with ethanol (originally placed onto valve 15) and water (valve 14) was deleted from the program. Instead, prior to synthesis, the C18 (light) SPE cartridge provided with the cassette was conditioned manually by slowly passing through 5 mL of ethanol (70%) and, subsequently, 5 mL of water (ultrapure grade).
- The labeling temperature in the reactor was set to 25 °C.
- Following the loading of the reactor content onto the SPE cartridge and the washing and drying of the cartridge, an additional elution step with 0.7 mL ethanol (40% *v*/*v* for [^68^Ga]Ga-TEoS-DAZA and 30% *v*/*v* for [^68^Ga]Ga-TMoS-DAZA) was included in the program. The ethanol was placed in a 3 mL Omnifix Luer Lock syringe (B. Braun), which was put onto valve 15.

Following these modifications, the radiosynthesis was performed as follows:

- The system dispensing unit eluted up to three ^68^Ga/^68^Ge generators placed in parallel via valves 7 and 8, using 5 mL of hydrochloric acid (0.1 M) per generator. The average starting activities were 1.8 ± 0.6 GBq.
- The eluate collected in the dispensing unit was slowly pushed over the cation exchange cartridge (PS-H^+^) provided from the reagent kit, via valves 8, 7, 6 and 1 into the waste. The cartridge was washed with 5 mL of water (valve 14) and dried via the mass flow controller (valve 11) with nitrogen.
- A total of 1.5 mL of 5 M sodium chloride solution (valve 7, provided in the reagent kit) was aspirated into the dispensing unit and slowly pushed over the cartridge via valve 2, thus transferring the ^68^Ga eluate into the reaction vessel (prefilled with 3 mL HEPES (1.5 M) from the reagent kit)
- The module allowed the reaction mixture to label for 10 min at 25 °C.
- The dispensing unit aspirated, subsequently passed the reaction mixture over the SPE cartridge via valves 4 and 5 and washed the cartridge twice with 10 mL of water (valve 14). The cartridge was dried via the mass flow controller (valve 11) with nitrogen.
- A total of 0.7 mL of ethanol (40% or 30% *v*/*v*, respectively) from valve 15 was aspirated and passed slowly over the SPE cartridge into the waste via valve 1.
- A total of 2 mL of ethanol (50% *v*/*v*) was aspirated from the vial (valve 9), which was provided along with the reagent kit, and was slowly passed over the SPE cartridge into the product vial (valve 3) via a cannula equipped with a 0.22 μm sterile filter provided in the reagent kit.
- A total of 15 mL of PBS buffer solution (valve 13) was aspirated by the dispensing unit and transferred into the product vial (valve 3).

For the ^68^Ga incorporation experiments of TEoS-DAZA, a cation exchange cartridge (PS-H^+^, Mackerey-Nagel) was preconditioned manually by slowly passing through 1.0 mL of hydrochloric acid (1.0 M) and, subsequently, 5.0 mL of water. A ^68^Ge/^68^Ga generator was eluted with 5.0 mL hydrochloric acid (0.1 M). The activity was collected on the preconditioned cation exchange cartridge and eluted into the reaction vial with sodium chloride solution (1.3 mL, 5.0 M, containing 32.5 µL of 5.5 M hydrochloric acid) [34]. The fresh ^68^Ga eluate was diluted with sodium chloride solution (5.0 M) to a total volume of 2.0 mL. Aliquots of 65 µL (50–55 MBq) were drawn and added to vials containing HEPES buffer solution (80 µL, 1.5 M, pH 4.5) or acetate buffer (120 µL, 1.0 M, pH 4.5), a respective aliquot of freshly prepared TEoS-DAZA stock solution (2–400 µL of a 5 µg/mL solution, *n* = 5) and water (0–438 µL) to ensure an identical total volume (585 µL) of all samples. The pH of the labeling solutions was 3.5 ± 0.1. Following the addition of ^68^Ga eluate, the samples were shaken briefly and left to stand for 10 min at room temperature. Subsequently, the percentage of non-chelated ^68^Ga(III) ions (hereinafter referred to as “free ^68^Ga”) in each sample was determined via radio TLC (0.1 M sodium citrate, silica gel 60 plates).

### 2.3. Radiolabeling of DEoS-DAZA and In Vitro Stability Determination

TEoS-DAZA (250 µg) was dissolved in 200 µL ethanol and 50 µL of hydrochloric acid (1.0 M) and diluted with 3 mL of HEPES buffer solution (1.5 M). The sample was heated to 100 °C for 10 min and then allowed to cool down to room temperature. HPLC analysis revealed that the sample contained 48% of DEoS-DAZA, 24% of EoS-DAZA and almost no detectable amounts of TEoS-DAZA. The complete sample was combined with 200 MBq of fresh ^68^Ga eluate and labeled for 10 min at room temperature. HPLC analysis showed that the sample contained 43% free ^68^Ga, 52% [^68^Ga]Ga-DEoS-DAZA and 5% [^68^Ga]Ga-TEoS-DAZA. The reaction mixture was passed over a C18 (light) SPE cartridge (preconditioned manually as described in 2.2) and washed with 5 mL of water. [^68^Ga]Ga-DEoS-DAZA (50 MBq) was eluted with 1.0 mL ethanol (30%, *v*/*v*). The RCP was 96.5%, as determined via HPLC (injection volume 10 µL, 500 kBq) and via radio TLC (5 µL spot, 250 kBq).

The sample of [^68^Ga]Ga-DEoS-DAZA in ethanol (30%, 1.0 mL, 50 MBq) was combined with PBS (0.5 mL) and human serum (0.7 mL) and incubated at 37 °C for 100 min. For radio TLC analysis, the plates were spotted directly with the solution. For radio HPLC analysis, an aliquot of the sample was passed over a preconditioned C18 (light) SPE cartridge. Immediately afterwards, 0.8 mL of ethanol (70% *v*/*v*) was passed over the cartridge as well and combined with the eluate. The cartridge and the activity of the eluate were measured, and the eluate was used for HPLC analysis.

### 2.4. Quality Control

The percentage of free ^68^Ga and of the by-products [^68^Ga]Ga-DEoS-DAZA and [^68^Ga]Ga-DMoS-DAZA, respectively, and the identity of [^68^Ga]Ga-TEoS-DAZA and [^68^Ga]Ga-TMoS-DAZA, respectively, were determined with radio HPLC using an eurosphere 125 × 4 mm column (100–5 C18, Knauer) and the following gradient: 0.0–2.5 min 97.0% A, 2.5–10.0 min 97.0% A → 0.0% A, 10.0–13.0 min 0.0% A, 13.0–13.05 min 0.0% A → 97.0% A, 13.05–16.0 min 97.0% A, A being water/trifluoroacetic acid (99.9/0.1 *v*/*v*) and B being acetonitrile/trifluoroacetic acid (99.9/0.1 *v*/*v*). Additionally, the percentage of free ^68^Ga was determined via radio TLC using silica gel 60 plates on aluminum; eluent: aqueous sodium citrate solution (0.1 M). The percentage of ^68^Ga in colloidal form was determined as described in the Ph. Eur. monograph for ^68^Ga-Edotreotide, using glass fiber plates (ITLC-SG) and 1/1 methanol/ammonium acetate (77 g/L) as the eluent [16]. A radio TLC scanner from Elysia Raytest (miniGita) was used for the examination of the TLC plates. The maximum content of HEPES was verified according to the method described in the Ph. Eur. monograph for ^68^Ga-Edotreotide, using a HEPES reference solution of 13.3 µg/mL [35]. Due to renovations of the gas supply, the ethanol content could not be measured via gas chromatography, as is required in the Ph. Eur. Instead, the osmolality of the samples was determined using a Semi-Micro Osmometer K-7400 (Knauer GmbH, Berlin, Germany). Using different samples of predetermined ethanol content, we performed a calibration to calculate the ethanol content of the product solutions from the osmolality values.

### 2.5. Octanol–Water–Partition Coefficient

[^68^Ga]Ga-TEoS-DAZA and [^68^Ga]Ga-TMoS-DAZA were produced by means of the aforementioned radiolabeling procedure and subsequently diluted with additional PBS to realize an ethanol content of max. 0.5% (*v*/*v*). From this solution, 2 mL, containing approximately 1 MBq of the respective radiotracer, was added to 2 mL of octanol in a centrifugal tube. After vigorous mixing of the suspension for 2 min at room temperature with a vortexer (1500 cpm), the vials were centrifuged at 685 rcf (3500 rpm) for 5 min. Six aliquots were drawn from the n-octanol layer (100 μL) and from the aqueous layer (300 µL), respectively, and measured in a gamma counter. The experiment was repeated five times for both radiotracers, respectively.

### 2.6. Biodistribution Studies and In Ovo PET Imaging

Biodistribution studies were performed on ostrich embryos on developmental day (DD) 37. A detailed description of the procedure of the artificial incubation and preparation of the embryos in the shell has been published previously [29]. In short, on DD 37, the embryos were immobilized using isoflurane (4%, 1 h), and a 4 × 2 cm piece of the calcified outer eggshell was removed with a rotating cutter, while keeping the inner shell membrane intact. Candling identified proper vessels of the chorion-allantois-membrane, enabling safe puncturing of a vein using a 30 G needle. Subsequently, the ostrich egg was placed on the PET/CT scanner, and tracer application via i.v. injection was performed at the same time that dynamic PET image acquisition was started. Imaging was performed with a standard PET/CT scanner (Biograph mCT40, Siemens, Erlangen, Germany) used in clinical routine examinations in humans. The scan protocol featured dynamic list mode acquisition for 60 min comprising six 10 s frames, three 20 s frames, six 30 s frames and eleven 5 min frames. Visual and quantification analyses of tracer distributions were performed using appropriate volume-of-interest (VOI) measurements of maximum and mean activity concentration. Additionally, differences in tracer uptake were shown by comparing standardized uptake values (SUVpeak) over time. Following the imaging procedure, the embryos were sacrificed via the i.v. injection of pentobarbital (1.0 mL) and subsequent decapitation. For the ex vivo quantification of activity distribution, blood, egg yolk and organs were collected and weighed. The activity of the samples was measured using a gamma counter (ISOMED 2100, NUVIA Instruments, Dresden, Germany) or, in the case of samples containing >0.2 MBq in total, a dose calibrator (ISOMED 2010, NUVIA Instruments, Dresden, Germany). As per national and international legislation, bird embryo studies do not qualify as an animal research study, as long as the embryos do not hatch [36,37,38]. Registration took place with the Office for Consumer Protection of the Thuringia State, registration number 22-2684-04-02-114/16.

## 3. Results

### 3.1. Precursor Stability during Radiolabeling

**Figure 1:**
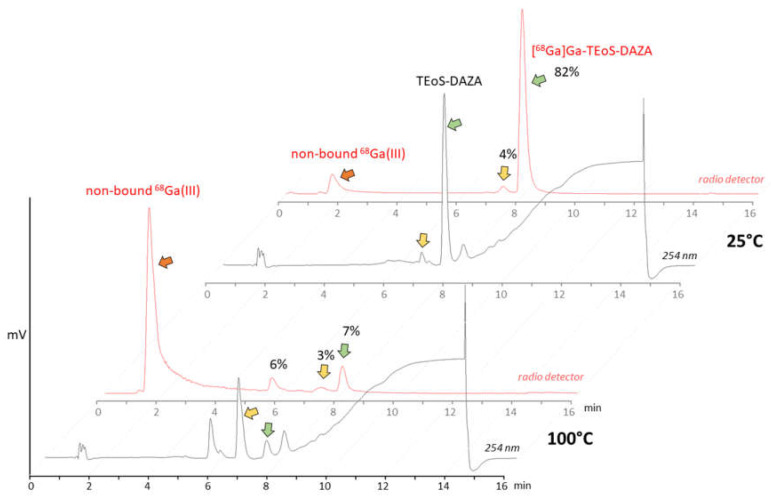
Top: HPLC analysis of precursor TEoS-DAZA in HEPES after 10 min at 25 °C (black line, UV/Vis channel) and analysis of the ^68^Ga labeling mixture at 25 °C (red line, radio detector channel). TEoS-DAZA (7.6 min) and its corresponding ^68^Ga complex (8.0 min) are indicated by green arrows. Bottom: HPLC analysis of TEoS-DAZA in HEPES after 5 min at 100 °C (black line) reveals the decomposition of the precursor (green arrow) into a major by-product (yellow arrow, 6.6 min), among other things. The corresponding ^68^Ga labeling mixture (red line) shows a high percentage of free ^68^Ga (red arrow) and only 7% of [^68^Ga]Ga-TEoS-DAZA (green arrow).

The initial test batch production of [^68^Ga]Ga-TEoS-DAZA on the automated synthesizer followed a standard protocol for ^68^Ga radiolabeling, using purified and concentrated ^68^Ga eluate from a ^68^Ge/^68^Ga generator [23]. HEPES buffer and a radiolabeling temperature of 100 °C were used. However, under these conditions, we observed unsatisfactory radiolabeling yields and low radiochemical purity. In-process radio HPLC analyses from the reaction vessel revealed high amounts of free ^68^Ga, while [^68^Ga]Ga-TEoS-DAZA made up only 7% of the total activity (Figure 1). The cause for this low radiochemical yield (RCY) was a rapid decomposition of the precursor TeoS-DAZA (7.4 min) in HEPES buffer at 100 °C, resulting in an unknown major by-product (6.6 min), as revealed by HPLC analysis (Figure 1). Besides the high amounts of free ^68^Ga, two radiolabeled by-products at 5.8 min and 7.4 min were identified in the corresponding radiolabeling mixture (Figure 1, yellow arrow). The same observation was made when using acetate buffer instead of HEPES.

**Figure 2:**
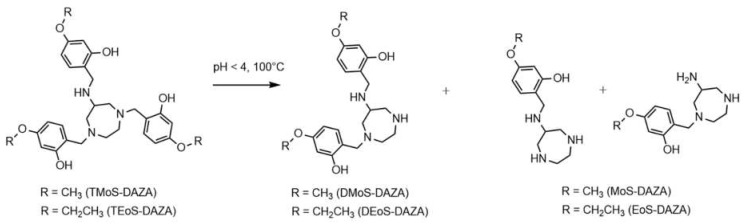
Structure of DEoS-DAZA and DMoS-DAZA (di-alkylated), respectively, and EoS-DAZA and MoS-DAZA (mono-alkylated), respectively, which are formed under acidic conditions. DEoS-DAZA and DMoS-DAZA are penta-dentate chelators that bind to ^68^Ga as well, thereby forming a radioactive impurity in the final product solutions.

Consequently, the radiolabeling was performed at 25 °C, which prevented precursor decomposition and produced [^68^Ga]Ga-TEoS-DAZA in satisfying yields (Figure 1). However, regardless of the temperature reduction, about 4% of the unidentified radiolabeled by-product at 7.4 min was still present in the reaction mixtures and in the final formulation. Since we found that all stock solutions of TEoS-DAZA contained 3–6% of the decomposition product at 6.6 min, even if freshly prepared at 25 °C (Figure 1), it seemed self-evident that this impurity was responsible for producing the radiolabeled by-product at 7.4 min. To verify this hypothesis and to identify the structure of the by-product, a sample of TEoS-DAZA was heated in HEPES and subsequently purified via reversed phase column chromatography. ESI-MS analysis of the fraction containing the by-product at 6.6 min revealed the structure to be a dialkylated DAZA fragment, hereinafter referred to as DEoS-DAZA, while another minor decomposition product at 5.6 min was identified as the monoalkylated species EoS-DAZA (Figure 2, Figures S1 and S2). Due to the very small amounts isolated, ^13^C{^1^H} NMR spectroscopic investigations of DEoS-DAZA and EoS-DAZA were not possible; however, ^1^H NMR spectroscopic investigations of the fraction containing DEoS-DAZA further clarified the structure. The methylene and methyl protons of the ethoxy group appearing as multiplets (Figure S3) indicate a non-symmetric substitution of the benzylic arms at the azacycle, as shown in Figure 2. HPLC analysis of the DEoS-DAZA fraction showed a single peak, further indicating that only the unsymmetrically substituted isomer of DEoS-DAZA was isolated. In contrast, the ^1^H NMR spectrum of EoS-DAZA was dominated by signals attributable to HEPES, so a clear interpretation of the spectrum was not possible. However, the isolated fraction of EoS-DAZA showed a double peak in the HPLC, indicating that both isomers, as presented in Figure 2, were likely formed.

By labeling DEoS-DAZA with ^68^Ga, we could verify that the radioactive by-product at 7.4 min is indeed [^68^Ga]Ga-DEoS-DAZA. Since DEoS-DAZA is a penta-dentate chelator, rapid demetallation of the ^68^Ga complex can be expected to occur under in vivo conditions. Indeed, in vitro stability measurements of [^68^Ga]Ga-DEoS-DAZA showed fast demetallation, while [^68^Ga]Ga-TEoS-DAZA is known to be stable in vitro [1].

**Figure 3:**
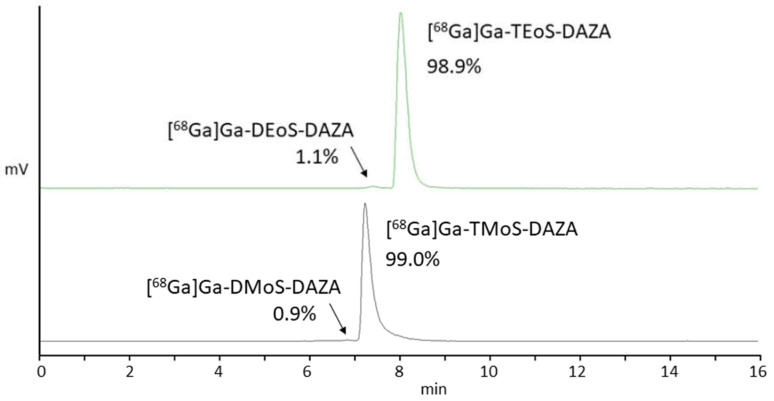
Representative radio HPLC chromatograms of [^68^Ga]Ga-TEoS-DAZA (green, top) and [^68^Ga]Ga-TMoS-DAZA (black, bottom) in the final formulation after SPE purification. Arrows point to the respective by-products [^68^Ga]Ga-DEoS-DAZA and [^68^Ga]Ga-DMoS-DAZA.

All in all, we observed similar results when radiolabeling TMoS-DAZA. Given that its structure is similar to TEoS-DAZA (apart from the methoxy instead of the ethoxy substitution), it is not surprising that TMoS-DAZA shows similar acidic lability and therefore requires labeling at 25 °C. Likewise, the radiolabeled by-product with less retention compared to [^68^Ga]Ga-TMoS-DAZA can be observed as impurity in the radio detector (Figure 3).

### 3.2. Optimized, GMP-Compliant, Fully Automated Synthesis of [^68^Ga]Ga-TEoS-DAZA and [^68^Ga]Ga-TMoS-DAZA

**Figure 4:**
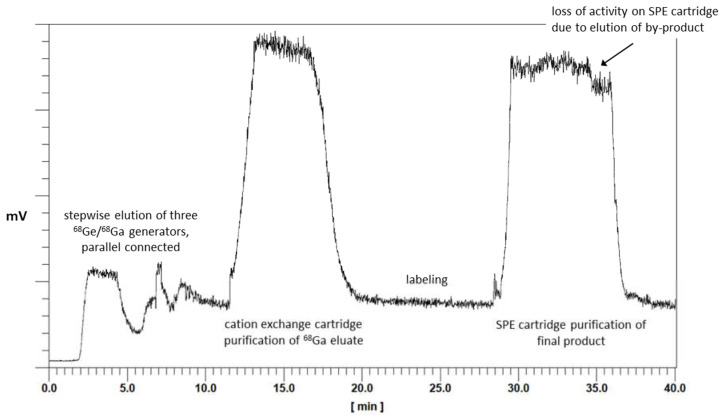
Chromatogram of activity sensors during the automated synthesis of a batch of [^68^Ga]Ga-TEoS-DAZA. The overall synthesis time is 40 min.

First, the GMP production was established for [^68^Ga]Ga-TEoS-DAZA, and the optimized parameters obtained therefrom were applied to the synthesis of [^68^Ga]Ga-TMoS-DAZA. Aiming to avoid precursor decomposition, the radiolabeling temperature was lowered to 25 °C while keeping the reaction time at 10 min. Under these conditions, the percentage of the by-product [^68^Ga]Ga-DEoS-DAZA in the reaction vessel was 4.3 ± 1.1% (*n* = 8). The by-product was found in similar percentages in the final product solution after C18-SPE purification using ethanol for elution (2 mL, 50% *v*/*v*). Therefore, the SPE purification procedure was modified. Prior to the elution of the main product, the cartridge was eluted with 0.7 mL of ethanol (40% *v*/*v*) into the waste container. Due to the lower content of ethanol, this fraction preferentially removes the less lipophilic [^68^Ga]Ga-DEoS-DAZA, while [^68^Ga]Ga-TEoS-DAZA, for the most part, remains on the cartridge. Subsequently, [^68^Ga]Ga-TEoS-DAZA is eluted into the final vial with ethanol (2 mL, 50% *v*/*v*). The synthesis was performed identically for [^68^Ga]Ga-TMoS-DAZA, except for the modification that 30% (*v*/*v*) ethanol (0.7 mL) was used for the elution of the by-product [^68^Ga]Ga-DMoS-DAZA. By this procedure, the content of the by-product in the final product solution was reproducibly lowered to 1.9 ± 0.9% for [^68^Ga]Ga-TEoS-DAZA and 1.3 ± 0.6% for [^68^Ga]Ga-TMoS-DAZA (Table 1). The two-stage, fractionated elution with the ethanol of different concentrations can be monitored on the activity chromatogram during synthesis. The first fraction of ethanol (40% or 30% *v*/*v*, respectively) causes a loss of total activity on the SPE cartridge of roughly 10% (Figure 4).

**Figure 5:**
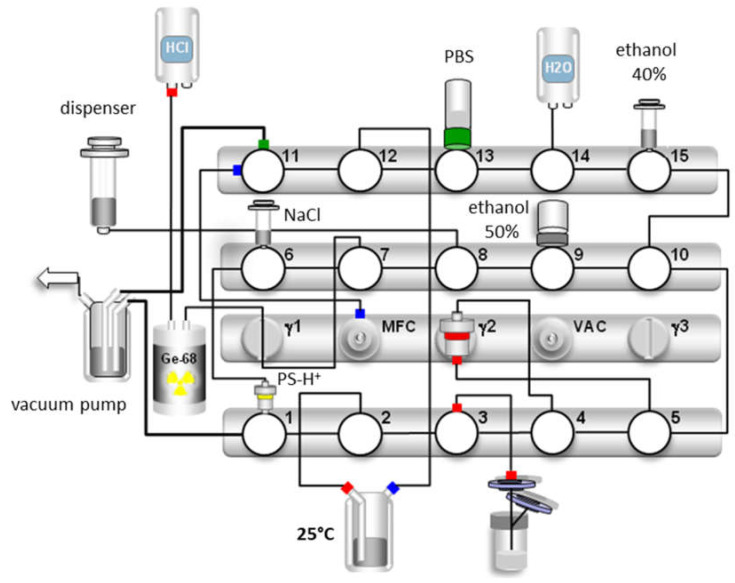
Synthesis scheme for [^68^Ga]Ga-TEoS-DAZA. For the production [^68^Ga]Ga-TMoS-DAZA, the synthetic procedure is identical, with the exception that the elution of the by-product from the SPE cartridge is carried out with 0.7 mL of ethanol 30% (*v*/*v*) instead of 40%, on valve 15.

The GMP productions of [^68^Ga]Ga-TEoS-DAZA and [^68^Ga]Ga-TMoS-DAZA were established and routinely performed on a GRP synthesizer (Scintomics, Figure 5). Prior to the synthesis, an aliquot of the precursor (1 µg/µL) was dissolved in a 5/1 mixture of ethanol and hydrochloric acid (1.0 M). Immediately after the preparation, a stock solution of TEoS-DAZA contained 2.5% of DEoS-DAZA, a content that rose to 4–5% after 2 weeks if the stock solution was kept at −24 °C, indicating that, if necessary, the precursor solutions may be stored in a freezer for several days and still provide successful radiolabeling (Figure S4). However, for GMP production, we recommend preparing a fresh stock solution. Depending on the starting activity, the optimal precursor amount was between 80 µg (up to 1.5 GBq) and 120 µg (up to 3 GBq). While different amounts of the precursor have no influence on the percentage of the by-product, the percentage of free ^68^Ga in the final product was out of specification (3.0 ± 1.4%) when using 80 µg of TEoS-DAZA for starting activities of ca. 3 GBq, whereas, for starting activities of ca. 1.5 GBq, the percentage of free ^68^Ga was within specification limits (0.2 ± 0.3%). In contrast, when using 120 µg of the precursor, no out-of-specification event occurred, even when using starting activities of ca. 3 GBq. Therefore, we decided to use 120 µg of the precursor for a standard production to maximize the percentage of ^68^Ga incorporation and, therefore, to reach the highest possible specific activity and RCY. The decay-corrected RCY of the GMP productions was 58 ± 10% (*n* = 6) for [^68^Ga]Ga-TEoS-DAZA and 53 ± 13% (*n* = 5) for [^68^Ga]Ga-TMoS-DAZA.

Previous experiments on the ^68^Ga incorporation depending on TEoS-DAZA concentration showed incomplete incorporation even after significantly increasing the precursor concentration (Figure S5). For example, in HEPES, the percentage of ^68^Ga incorporation was raised from 65 ± 20%, using 0.25 µg of the precursor, to only 80 ± 11%, by using an eightfold amount (2 µg or 3.4 µg/mL) of the precursor. The extrapolation of this increasingly flat curve results in a theoretically required amount of ca. 40 µg TEoS-DAZA for the complete ^68^Ga chelation of a 50 MBq aliquot, which equals a specific activity of 1.2 MBq/µg. However, batches of [^68^Ga]Ga-TEoS-DAZA produced on the GRP synthesizer using a complete ^68^Ga eluate and 120 µg of the precursor yielded the tracer in specific activities of 6.9 ± 2.3 MBq/µg (Table 1). Thus, while the ^68^Ga incorporation experiments in general reflect a rather low efficiency of the ^68^Ga chelation properties of TEoS-DAZA, the effectiveness of chelation in a routine clinical production is, in fact, higher.

### 3.3. Quality Control

Batches of [^68^Ga]Ga-TEoS-DAZA and [^68^Ga]Ga-TMoS-DAZA reproducibly yielded formulations that complied with the specifications on ^68^Ga tracers and, in general, sterile injection solutions, which are required according to the European Pharmacopeia (Ph. Eur.) [39]. The published monograph on ^68^Ga-Edotreotide was used for reference to define product specifications and quality control (QC) methods [35]. Wherever feasible, the methods described in the Ph. Eur. were used for QC (Table 1).

In addition to free ^68^Ga and ^68^Ga in colloidal form, [^68^Ga]Ga-DEoS-DAZA or [^68^Ga]Ga-DMoS-DAZA, respectively, constitute a third possible impurity that may lower the RCP of the final products. Since the instability of [^68^Ga]Ga-DEoS-DAZA in serum was shown, the decomplexation of these by-products under in vivo conditions can be expected to mimic the unspecific distribution of free ^68^Ga, thus lowering image quality. While the content of free ^68^Ga, [^68^Ga]Ga-DEoS-DAZA and [^68^Ga]Ga-DmoS-DAZA can be quantified with radio HPLC, we discovered that the TLC method, as described in the Ph. Eur. for the determination of ^68^Ga in colloidal form, is inhibited by two facts. Firstly, instead of giving a sharp peak, the radiotracers exhibit a strong peak tailing while migrating to the front. The tailing is more pronounced for [^68^Ga]Ga-TEoS-DAZA than for [^68^Ga]Ga-TMoS-DAZA (Figures S7 and S9), indicating that increased molecular weight and/or lipophilicity could play a role. For ^68^Ga tracers that are based on very large molecules, such as exendin, similar difficulties due to strong retention or tailing have been reported before [40,41,42]. However, if the retention factor used to calculate the percentage of [^68^Ga]Ga-TEoS-DAZA and [^68^Ga]Ga-TMoS-DAZA, respectively, is assigned to Rf = 0.5–1.0, the signal separation to the ^68^Ga colloid at Rf = 0.0–0.2 is sufficient. Since the peak tailing extends to Rf = 0.2 in the case of [^68^Ga]Ga-TEoS-DAZA, a part of the tracer activity is not included in the calculation, meaning that the percentage of the ^68^Ga colloid is always overestimated, so no risk arises.

However, the second inhibition of the TLC method for the determination of ^68^Ga in colloidal form stems from the content of the by-products [^68^Ga]Ga-DEoS-DAZA and [^68^Ga]Ga-DMoS-DAZA in the final formulations. [^68^Ga]Ga-DEoS-DAZA remains, to a large extent, at the bottom of the TLC plate, along with the ^68^Ga colloid, while additionally migrating towards the front over the entire plate (Figure S11). This unusual chromatographic profile might stem from the disintegration of the penta-coordinate [^68^Ga]Ga-DEoS-DAZA under these TLC conditions. Consequently, the activity at Rf = 0.0–0.2 must be considered to be the sum of the ^68^Ga colloid and a substantial part of [^68^Ga]Ga-DEoS-DAZA or [^68^Ga]Ga-DMoS-DAZA, once again meaning that the colloid content is always overestimated. Therefore, for the quality control protocol, we set the limit for the activity of the bottom of the plate to ≤5.0% of the total activity, while the limit for the percentage of [^68^Ga]Ga-DEoS-DAZA or [^68^Ga]Ga-DMoS-DAZA (as determined via radio HPLC) was set to ≤3.0%. To compensate for these additional impurities, the limit of free ^68^Ga was lowered from ≤2.0% (as specified in the ^68^Ga-Edotreotide monograph) to ≤1.0% (Table 1). Therefore, in total, the RCP of the radiotracers is ensured to be ≥96.0%, as determined via radio HPLC, and ≥95.0%, as determined via the radio TLC method for the determination of the colloid.

### 3.4. Biodistribution Studies and logP

**Figure 6:**
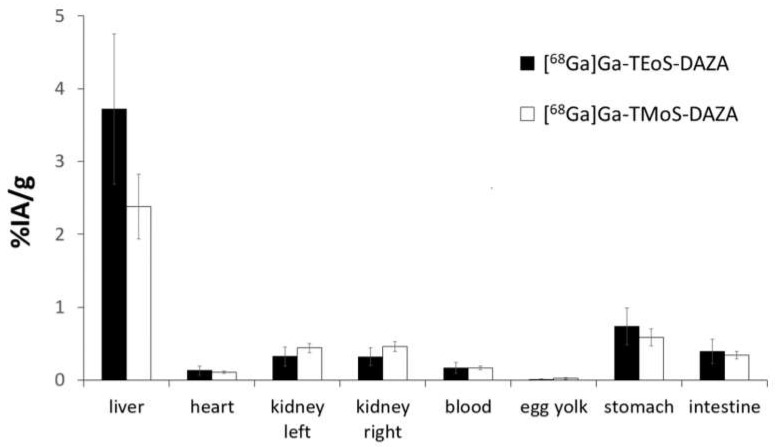
Biodistribution of [^68^Ga]Ga-TEoS-DAZA and [^68^Ga]Ga-TMoS-DAZA in ostrich embryos (*n* = 7), given as mean values of %IA/g. The standard deviation is indicated by error bars.

The results of the biodistribution are presented as the percentage of injected activity per gram of tissue (%IA/g); values are expressed as the mean ± SD (Figure 6). A group of seven embryos was investigated for [^68^Ga]Ga-TEoS-DAZA and [^68^Ga]Ga-TMoS-DAZA, respectively. Notably, the uptake in the liver is highest compared to all other tissue. Since [^68^Ga]Ga-TEoS-DAZA exhibits a higher lipophilicity compared to [^68^Ga]Ga-TMoS-DAZA, as can be inferred from the higher logP value (Table 2) as well as the stronger retention on the HPLC column (Figure 3), it is not surprising to find that [^68^Ga]Ga-TEoS-DAZA exhibits a higher uptake in the liver (3.72 ± 1.03%IA/g) than [^68^Ga]Ga-TMoS-DAZA (2.62 ± 1.09%IA/g). Accordingly, the uptake in the kidneys was slightly higher for [^68^Ga]Ga-TMoS-DAZA (0.48 ± 0.16%IA/g) than for [^68^Ga]Ga-TEoS-DAZA (0.33 ± 0.13%IA/g), although, for both organs, a *t*-test revealed that the difference in uptake was not statistically significant. Since ostriches do not have a gall bladder, the activity in the intestines is used as a parameter for hepatobiliary excretion. The intestinal activity at 60 min p.i. was comparable for both radiotracers, indicating a certain percentage of hepatobiliary excretion via the gall tree. Surprisingly, the stomach showed a rather high tracer accumulation as well.

### 3.5. PET Imaging

**Figure 7:**
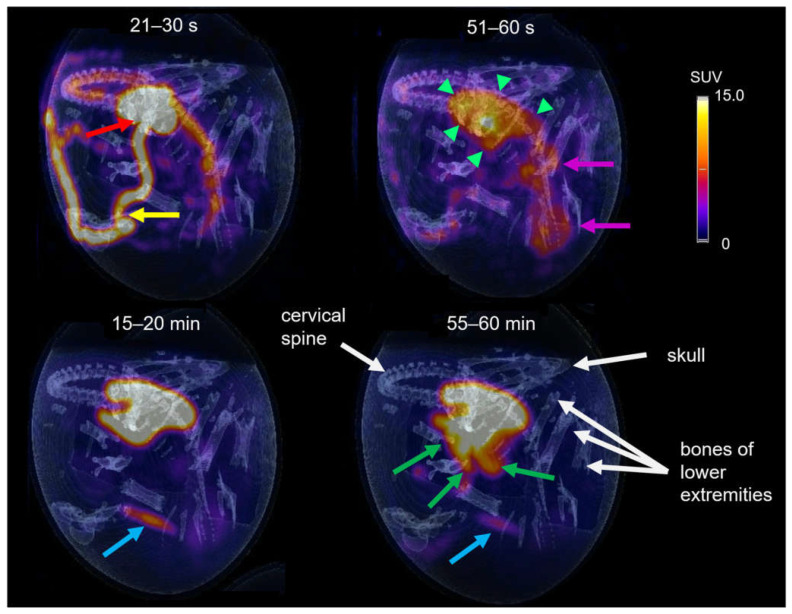
PET/CT of [^68^Ga]Ga-TMoS-DAZA in an ostrich embryo at different time frames. At early time points the vitelline vein (yellow arrow) and the heart (red arrow) are visible, as well as early accumulation in the liver (short green arrows) and kidneys (pink arrows). After 15–20 min a weak activity accumulation in the cloaca (blue arrow) can be observed, while excretion into the duodenum (green arrows) is visible at 55–60 min. White arrows point to osseous structures.

The tracer distribution in ovo over time was determined via dynamic PET imaging. Representative PET/CT images of [^68^Ga]Ga-TMoS-DAZA at defined time points are presented in Figure 7. In the first frame (21–30 s post injection), the injected activity is located within the vitelline vein (yellow arrow) and the heart (red arrow). At 51–60 s, tracer accumulation within the liver is observed (green arrows). Additionally, the characteristic shape of the barbell-shaped kidneys is visible due to a weak renal accumulation (pink arrows). During the following 20 min, the activity accumulation in the liver intensifies, while unspecific renal activity decreases gradually. The blue arrow at 15–20 min points to a pararenal tubular accumulation with a sudden onset from 15 min p.i. and a subsequent slow decrease in intensity (blue arrow). This corresponds to renal excretion via the ureter into the cloaca. At 40 min p.i., the excretion of the activity from the liver into the intestinal tract begins. At 55–60 min, the activity in the intestines is visible as a diffuse shape, which corresponds to the sinuous structure of the small intestinal loops (green arrows). In addition, osseous structures are named for orientation purposes (white arrows).

**Figure 8:**
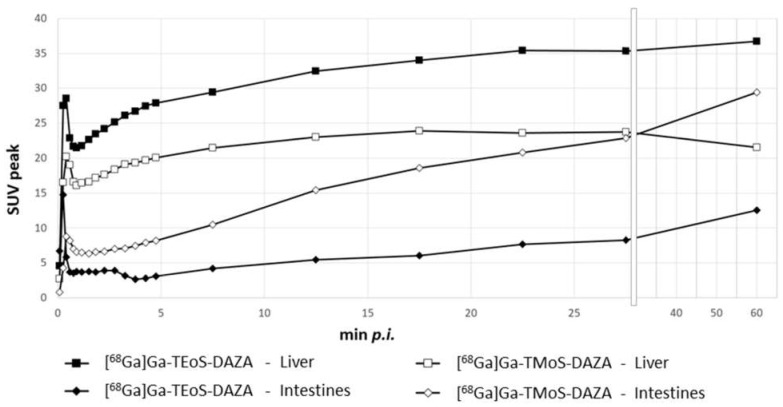
SUVpeak in the liver and the intestines for both ^68^Ga-tracers, respectively, given as the mean (*n* = 7). Error bars are omitted for clarity. From the 28th minute onwards, the x axis is compressed.

Based on dynamic PET images, as presented in Figure 7, uptake values were calculated and are given as the SUVpeak (Figure 8). The curves for the liver and intestine VOIs show a distinct peak within the first minute after tracer application, representing overlying blood pool activity. This artifact is more pronounced in liver VOIs caused by the close proximity of the heart and liver. Subsequently, a steady accumulation of the tracer in the liver can be observed up to 5 min p.i. This trend is followed by a flattened curve showing a slight increase for [^68^Ga]Ga-TEoS-DAZA throughout the entire 60 min and a slight decrease for [^68^Ga]Ga-TMoS-DAZA beginning at 17 min p.i. The lower liver SUVpeak for [^68^Ga]Ga-TMoS-DAZA is in accordance with the ex vivo biodistribution results and its lower lipophilicity.

The intestine activity for both tracers increased steadily over time, indicating the hepatobiliary excretion of activity from the liver. The intestine activity of [^68^Ga]Ga-TMoS-DAZA is notably higher than that for [^68^Ga]Ga-TEoS-DAZA and increases more significantly. This could indicate a faster hepatocellular transit time of the less lipophilic [^68^Ga]Ga-TMoS-DAZA, which is in accordance with the fact that while a high lipophilicity in general may increase the liver uptake of a tracer, it can slow the biliary excretion rate at the same time [43]. However, one has to keep in mind that the standard deviation is quite high, especially for [^68^Ga]Ga-TMoS-DAZA, and not all mean values were statistically significant for each time point between the two tracers. Furthermore, the PET findings are obviously not in accordance with the results of the ex vivo biodistribution study, which showed no significant difference in intestine activity between both tracers. For quantification from PET, the VOI was placed in the proximal duodenum close to the liver. Therefore, the SUVpeak reflects the local tracer concentration in a defined region of the organ, while the ex vivo measurements reflect the tracer concentration in the whole organ. More detailed evaluation procedures would be needed to more accurately validate the congruence between the two methods.

## 4. Discussion

Due to its higher liver uptake and logP value, [^68^Ga]Ga-TEoS-DAZA seemed to be the more promising candidate from early on. Therefore, detailed studies on precursor decomposition and the ^68^Ga incorporation experiments presented herein were performed only for this precursor. However, given their closely related chemical structure, similar results regarding stability and chelating efficiency can be expected for TMoS-DAZA.

In acidic reaction buffers at high temperatures, TEoS-DAZA shows a fast decomposition into the N-dealkylated species DEoS-DAZA and EoS-DAZA, indicating stepwise C-N-bond cleavage of the pendant arms. To our knowledge, this observation is quite unusual, as the N-dealkylation of amines usually requires the presence of chloroformates, enzymes or metal catalysts [44]. Since TEoS-DAZA is insoluble in water, stock solutions for radiolabeling were prepared in mixtures of ethanol and hydrochloric acid. This apparently already leads to a certain amount of N-dealkylation at room temperature, since DEoS-DAZA is a persistent impurity in all stock solutions. Since DEoS-DAZA, too, forms a ^68^Ga complex, its presence in the radiolabeling mixture leads to the reduced RCP of the final product.

In 2018, we reported ^68^Ga to be labeled by TEoS-DAZA and TMoS-DAZA at 100 °C within 5 min, which contradicts our results presented herein [1]. This discrepancy can be explained by the fact that, in 2018, the synthesis was performed manually by combining the ^68^Ga eluate, precursor and buffer in a reaction tube and then placing it in the heating unit of a synthesizer module. Since the reaction tubes do not fit very well into the heating unit, it is likely that the temperature transfer was quite inefficient. Therefore, we assume that the temperature within the reaction tubes rose rather slowly, resulting in less precursor decomposition within the first few minutes and ensuring sufficiently successful radiolabeling. Additionally, in 2018 an R&D grade HPLC equipped with a radio detector of a comparably low sensitivity was used. As a result, the by-products [^68^Ga]Ga-DEoS-DAZA and [^68^Ga]Ga-DMoS-DAZA were not detected at that time. Only after switching to a different automated synthesis module for the GMP validation which features an efficient preheating of the reaction unit and using a highly sensitive radio HPLC did we become aware of the radiolabeling difficulties.

Since the rapid precursor decomposition at high temperatures requires radiolabeling at room temperature, the radiolabeling efficiency is comparatively low. The reaction mixtures usually contain about 9% of free ^68^Ga, which indicates incomplete chelation and is reflected by the results of the ^68^Ga incorporation experiments as well. Consequently, the amount of precursor (120 µg) required for satisfying radiolabeling yields in the automated procedure is high compared to other precursors such as DOTATOC or PSMA, which are commonly used in amounts of 20–50 µg. Considering that TEoS-DAZA and TMoS-DAZA are small molecules and DOTATOC or PSMA are conjugated peptides of a high molar mass, the discrepancy in terms of molar quantities is even higher. To remain within the safety profile, we therefore limit the maximum volume of the applicable product solution per injection to 5 mL [^68^Ga]Ga-TEoS-DAZA or [^68^Ga]Ga-TMoS-DAZA, meaning that, per patient, no more than 40 µg of excess precursor is applied. We recommend an injection dose of 50–150 MBq per patient.

By introducing an additional SPE elution step, the content of the respective by-products—[^68^Ga]Ga-DEoS-DAZA and [^68^Ga]Ga-DMoS-DAZA—in the final formulation could be lowered to ≤3.0%, which is an acceptable level based on risk assessment. The content of the ^68^Ga colloid can be determined according to the Ph. Eur. Method; however, radiopharmacists must be aware that the by-products are also in part retained at the bottom of the TLC plate. The increase in the acceptance limit for the method to ≤5.0% reflects the sum of both impurities in sufficient approximation. In summary, [^68^Ga]Ga-TEoS-DAZA and [^68^Ga]Ga-TMoS-DAZA can be prepared in satisfying yields according to GMP guidelines, which means that a transition into the early clinical phase is now possible.

Both [^68^Ga]Ga-TEoS-DAZA and [^68^Ga]Ga-TMoS-DAZA exhibit specific and rapid liver uptake and excretion into the intestines, both in the in ovo model and in a first compassionate use [28]. However, both the ex vivo biodistribution studies and the PET quantification show that, in the in ovo model, substantial amounts of activity are retained in the liver, even at 60 min p.i., and the biliary excretion rate is comparably low. Currently, the in ovo model using ostrich embryos is still an experimental model; therefore, the transferability of the biodistribution results to mammals has not yet been conclusively validated. Some anatomical differences between birds and mammals are likely to influence the results. For example, ostriches do not have a urine bladder but instead excrete fluids and feces via the cloaca. Furthermore, in the embryonal state, additional deviating mechanisms influencing excretion kinetics and organ functions must be taken into account. For example, ostrich embryos handle fluid excretion via an external sac-like structure, called the allantois, rather than via the cloaca. Likewise, it can be assumed that the hepatobiliary-intestinal tract is not yet fully functioning at this development state. Nevertheless, while the biodistribution and pharmacokinetic results of [^68^Ga]Ga-TEoS-DAZA and [^68^Ga]Ga-TMoS-DAZA in ovo need to be carefully scrutinized and evaluated, a first compassionate use in man showed that the biliary excretion of [^68^Ga]Ga-TMoS-DAZA is indeed sufficient to perform gall tree diagnoses [28]. Therefore, the potential of these radiotracers for hepatobiliary PET imaging could be demonstrated.

To better understand the mechanism behind tracer uptake and excretion, transporter binding studies and in vivo metabolite analyses of these radiotracers should be performed. Additionally, the cause of the non-insignificant tracer uptake in the stomach should be examined. Furthermore, since TEoS-DAZA and TMoS-DAZA are novel phenolate-based heterocycles, thermodynamic and kinetic complex stability studies should be performed to allow for a better comparison with established chelators.

## 5. Patents

The precursors TEoS-DAZA and TMoS-DAZA and the ^68^Ga radiotracers are protected by patent DE102017129405A1.

## Figures and Tables

**Table 1:** Quality control specifications of [^68^Ga]Ga-TEoS-DAZA and [^68^Ga]Ga-TMoS-DAZA produced on the synthesizer.

		Product Specifications	[^68^Ga]Ga-TEoS-DAZA	[^68^Ga]Ga-TMoS-DAZA
		Yield	828 ± 285 MBq	752 ± 224 MBq
		Specific Activity	6.9 ± 2.3 MBq/µg	6.3 ± 1.9 MBq/µg
		Volume	15 ± 0.9 mL	
Quality Control	Method	Acceptance Criteria	Result	
Appearance	Visual inspection	Clear, colorless solution	Complies	
pH	Potentiometric Determination ^1^	6–8	7.6 ± 0.1	7.5 ± 0.1
Ethanol content	Osmolality measurement	≤10% (*v*/*v*)	5.1 ± 1.1%	5.1 ± 0.9%
HEPES content	TLC ^1^	≤200 µg/15 mL, intensity of test solution spot similar or less than reference solution	Complies	
Radionuclide identity	Half-life ^1^	62–74 min	68.2 ± 0.6 min	68.3 ± 0.4 min
Radionuclide identity	Gamma-ray Spectrometry ^1^	511 keV and 1077 keV	Complies	
Content of ^68^Ge (radionuclide purity)		≤0.001%	1 × 10^−5^ ± 0.3 × 10^−5^	1 × 10^−5^ ± 0.3 × 10^−5^
Content of free ^68^Ga	Radio HPLC ^1^	≤1.0%	0.2 ± 0.3%	0.3 ± 0.2%
Activity at the bottom of the TLC plate	Radio TLC ^1^, test for ^68^Ga colloid	≤5.0%	3.2 ± 1.4%	1.5 ± 0.6%
Content of [^68^Ga]-DEoS-DAZA or [^68^Ga]-DMoS-DAZA, respectively	Radio HPLC	≤3.0%	1.9 ± 0.9%	1.3 ± 0.6%
Bacterial endotoxins	LAL test ^1^	≤175 IU/V	0.6 ± 0.2 EU/mL	0.5 ± 0.0 EU/mL
Sterility	Sterility testing ^1^	Sterile	Complies	

^1^ All noted methods were performed in accordance with the requirements of the Eur. Ph. monograph of ^68^Ga-Edotreotide. Results are given as the mean ± SD (*n* = 8 for [^68^Ga]Ga-TEoS-DAZA and *n* = 5 for [^68^Ga]Ga-TMoS-DAZA).

**Table 2:** Octanol-water-partition coefficient (logP) of [^68^Ga]Ga-TEoS-DAZA and [^68^Ga]Ga-TMoS-DAZA, as determined via a shake-flask-method.

	logP
[^68^Ga]Ga-TEoS-DAZA	1.6 ± 0.1
[^68^Ga]Ga-TMoS-DAZA	0.9 ± 0.1

## Data Availability

Not applicable.

## Supplementary Materials

The following supporting information can be downloaded at: https://www.mdpi.com/article/10.3390/pharmaceutics14122695/s1, Figure S1: ESI-MS of DEoS-DAZA; Figure S2: ESI-MS of EoS-DAZA; Figure S3: ^1^H NMR spectrum of DEoS-DAZA (Methanol-d_4_, 400 MHz) along with detailed depictions of the regions containing the signals of the ethoxy groups at the benzylic pendant arms (CH_2_ moiety at ca. 4.0 ppm and CH_3_ moiety at ca. 1.4 ppm); Figure S4: Content of TEoS-DAZA in a 1 µg/µL stock solution of 5/1 ethanol/hydrochloric acid (1.0 M), kept at −24 °C; Figure S5: ^68^Ga incorporation (mean ± SD) of TEoS-DAZA in HEPES and acetate buffer at 25 °C, 10 min, pH 3.5, 50 ± 4 MBq per sample. From the third value onwards (0.085 µg/mL), the x axis is contracted; Figure S6: Radio TLC plate of a sample of [^68^Ga]Ga-TEoS-DAZA. Stationary phase: Silica gel 60 on aluminum; eluent: aqueous sodium citrate solution (0.1 M); Figure S7: Radio TLC plate of a sample of [^68^Ga]Ga-TEoS-DAZA. Stationary phase: glass fiber plate (ITLC-SG); eluent: methanol/ammonium acetate (77g/L) 1/1; Figure S8: Radio TLC plate of a sample of [^68^Ga]Ga-TMoS-DAZA. Stationary phase: Silica gel 60 on aluminum; eluent: aqueous sodium citrate solution (0.1 M); Figure S9: Radio TLC plate of a sample of [^68^Ga]Ga-TMoS-DAZA. Stationary phase: glass fiber plate (ITLC-SG); eluent: methanol/ammonium acetate (77g/L) 1/1; Figure S10: Radio TLC plate of a sample of [^68^Ga]Ga-DEoS-DAZA. Stationary phase: Silica gel 60 on aluminum; eluent: aqueous sodium citrate solution (0.1 M); Figure S11: Radio TLC plate of a sample of [^68^Ga]Ga-DEoS-DAZA. Stationary phase: glass fiber plate (ITLC-SG); eluent: methanol/ammonium acetate (77 g/L) 1/1.
