# Supplementary figures and images for: Automated GMP Production and Preclinical Evaluation of [68Ga]Ga-TEoS-DAZA and [68Ga]Ga-TMoS-DAZA
Source: Pharmaceutics. 2022 Dec 1;14(12):2695. doi: 10.3390/pharmaceutics14122695 (PMC9783202; doi:10.3390/pharmaceutics14122695)

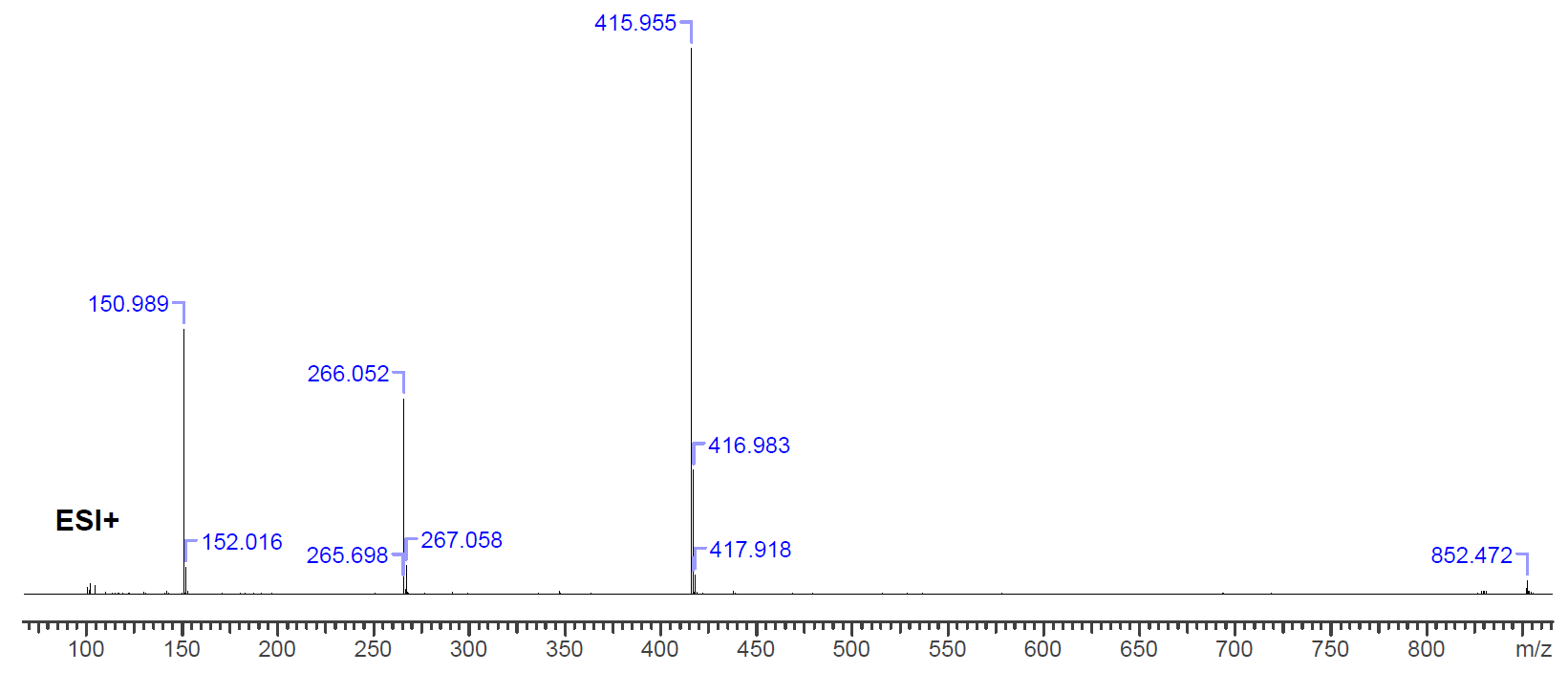

Supplement: Supplementary file 1 [file pharmaceutics-14-02695-s001.zip › Figure S1.tif]

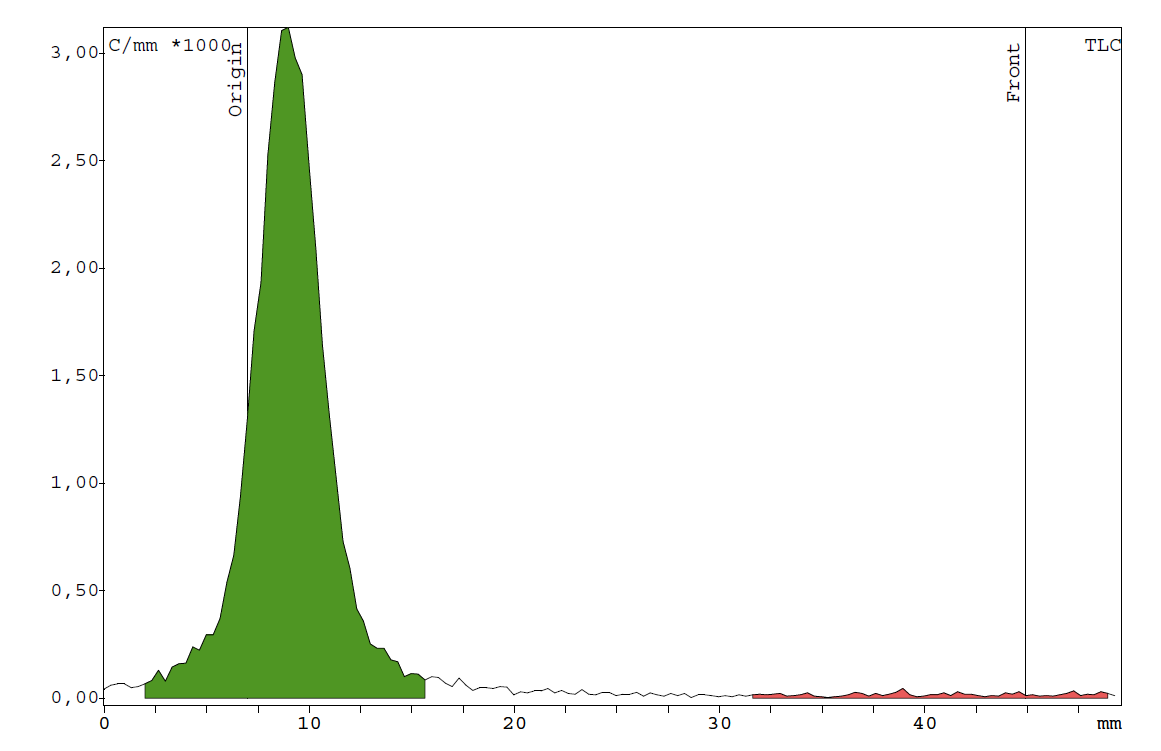

Supplement: Supplementary file 1 [file pharmaceutics-14-02695-s001.zip › Figure S10.png]

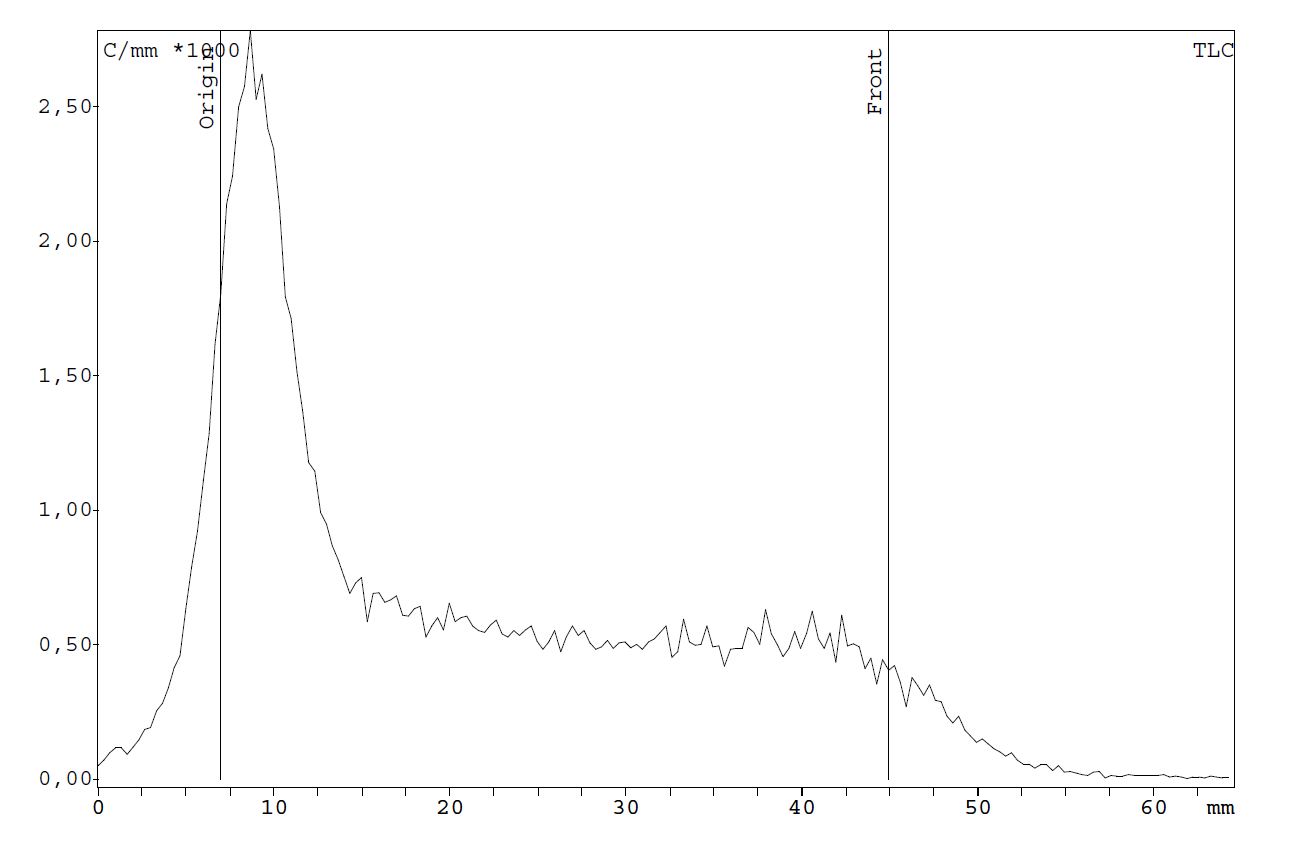

Supplement: Supplementary file 1 [file pharmaceutics-14-02695-s001.zip › Figure S11.png]

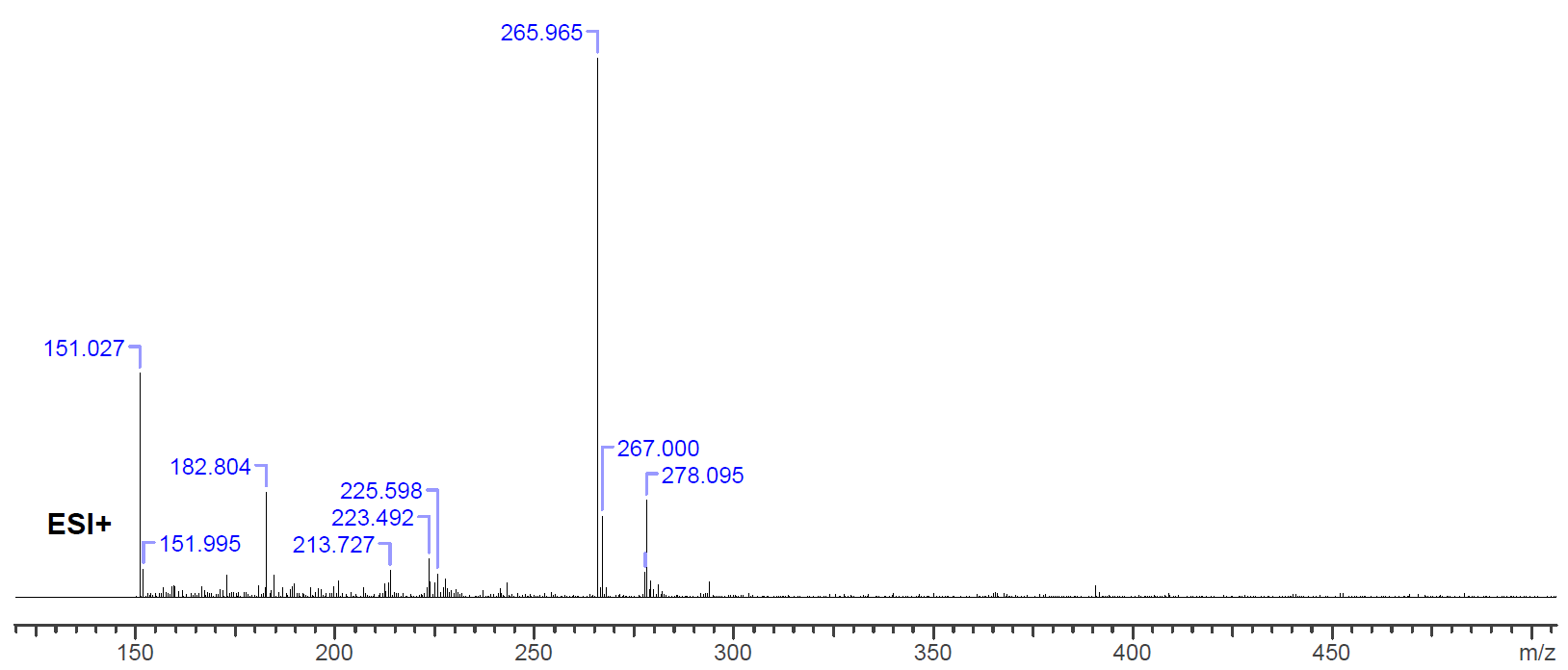

Supplement: Supplementary file 1 [file pharmaceutics-14-02695-s001.zip › Figure S2.tif]

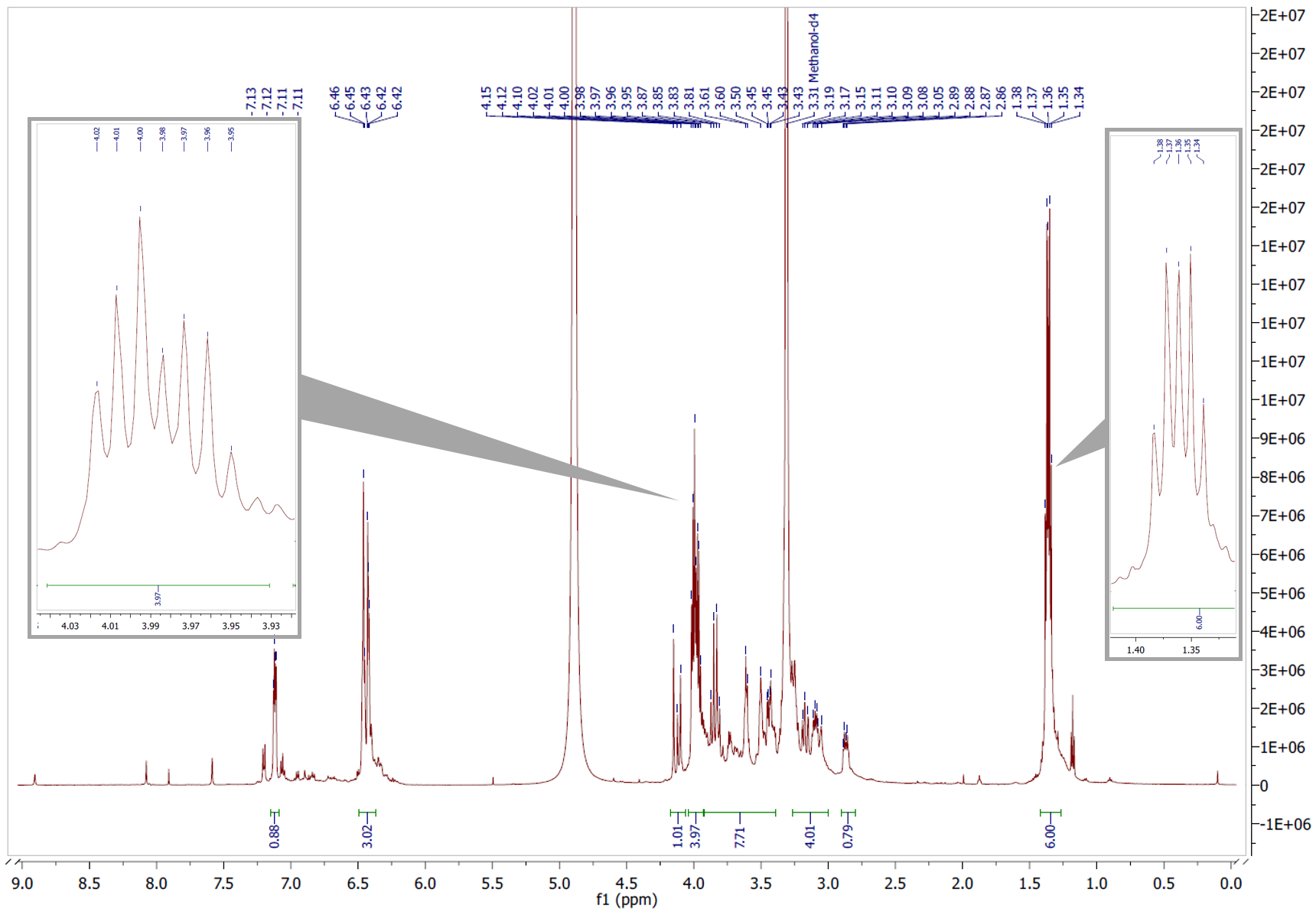

Supplement: Supplementary file 1 [file pharmaceutics-14-02695-s001.zip › Figure S3.tif]

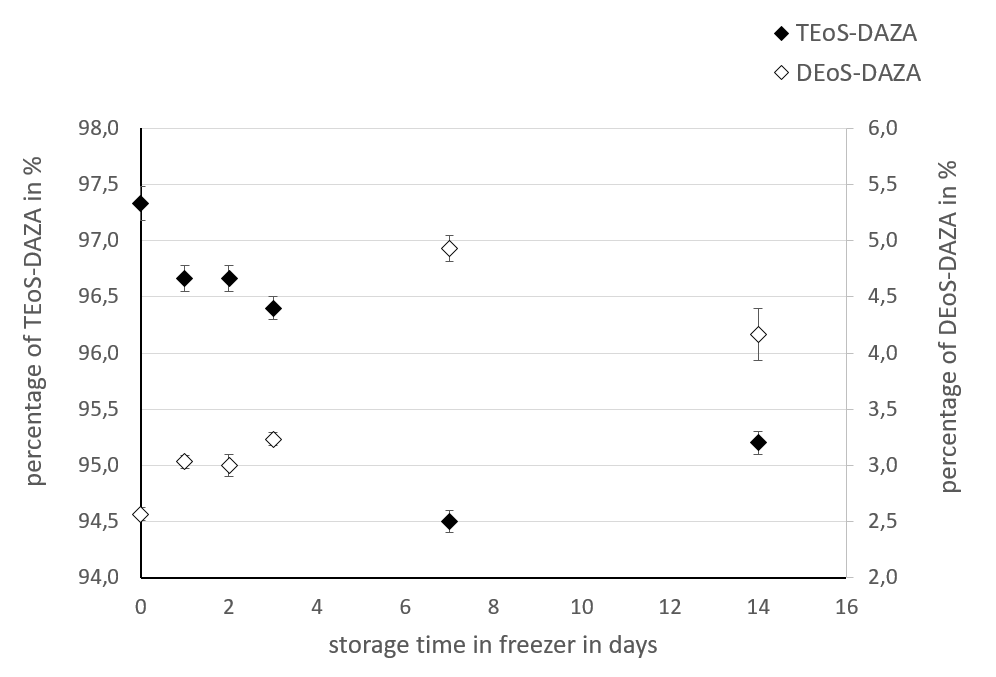

Supplement: Supplementary file 1 [file pharmaceutics-14-02695-s001.zip › Figure S4.png]

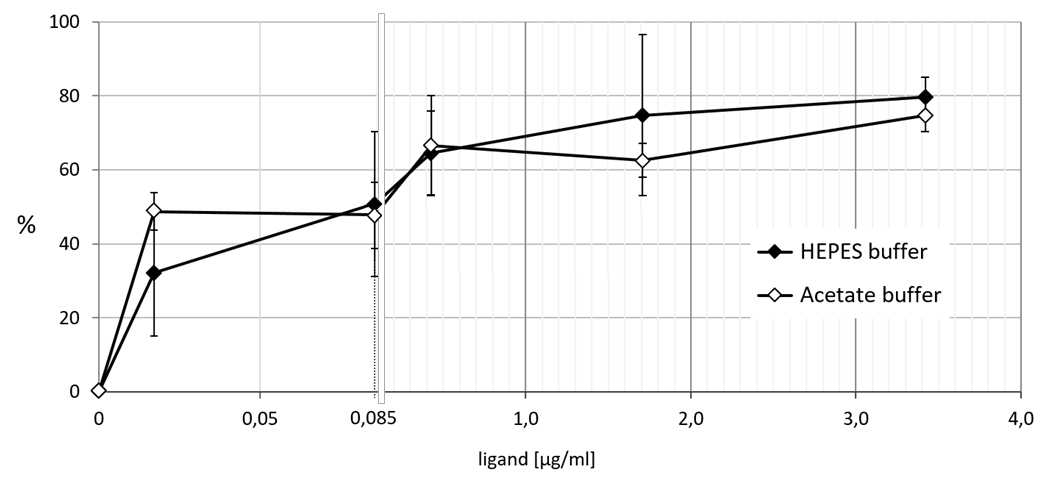

Supplement: Supplementary file 1 [file pharmaceutics-14-02695-s001.zip › Figure S5.tif]

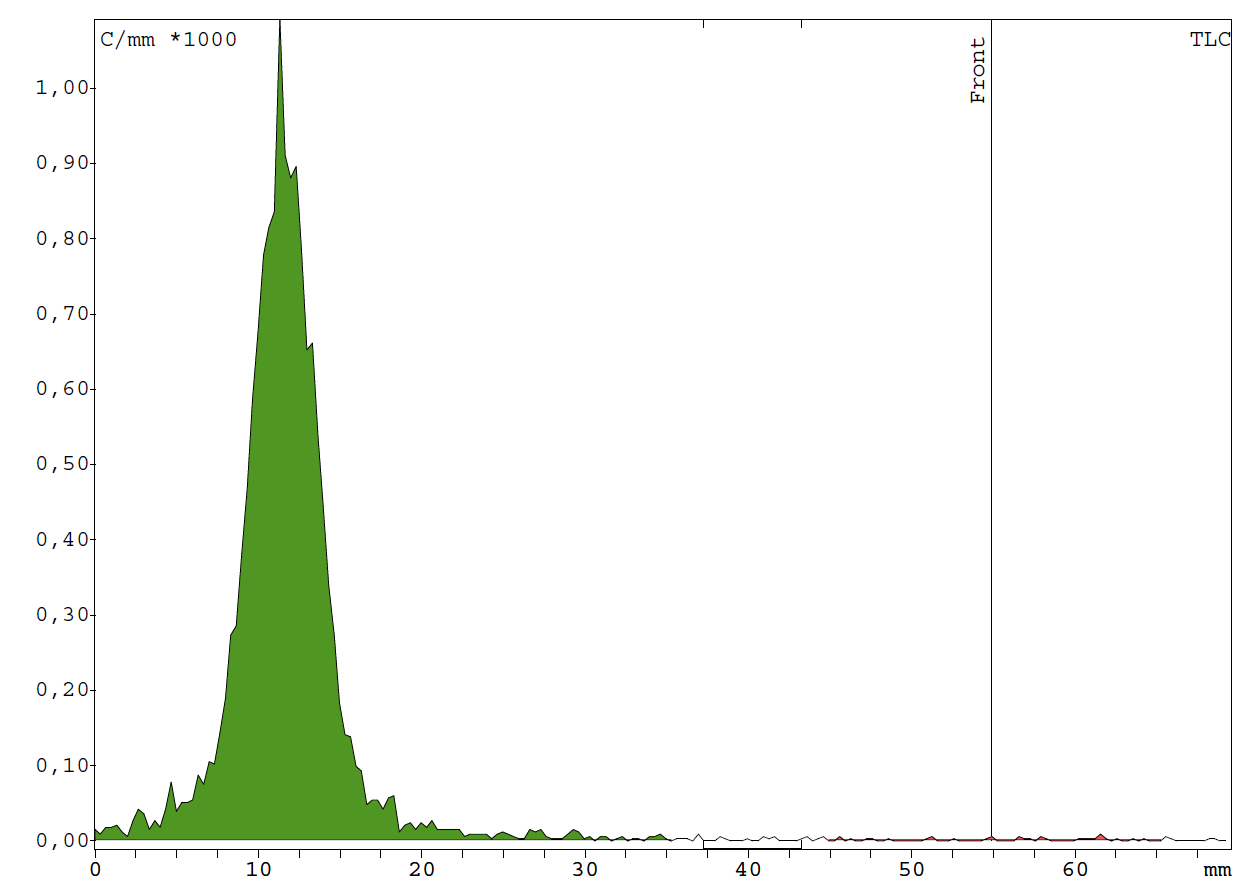

Supplement: Supplementary file 1 [file pharmaceutics-14-02695-s001.zip › Figure S6.png]

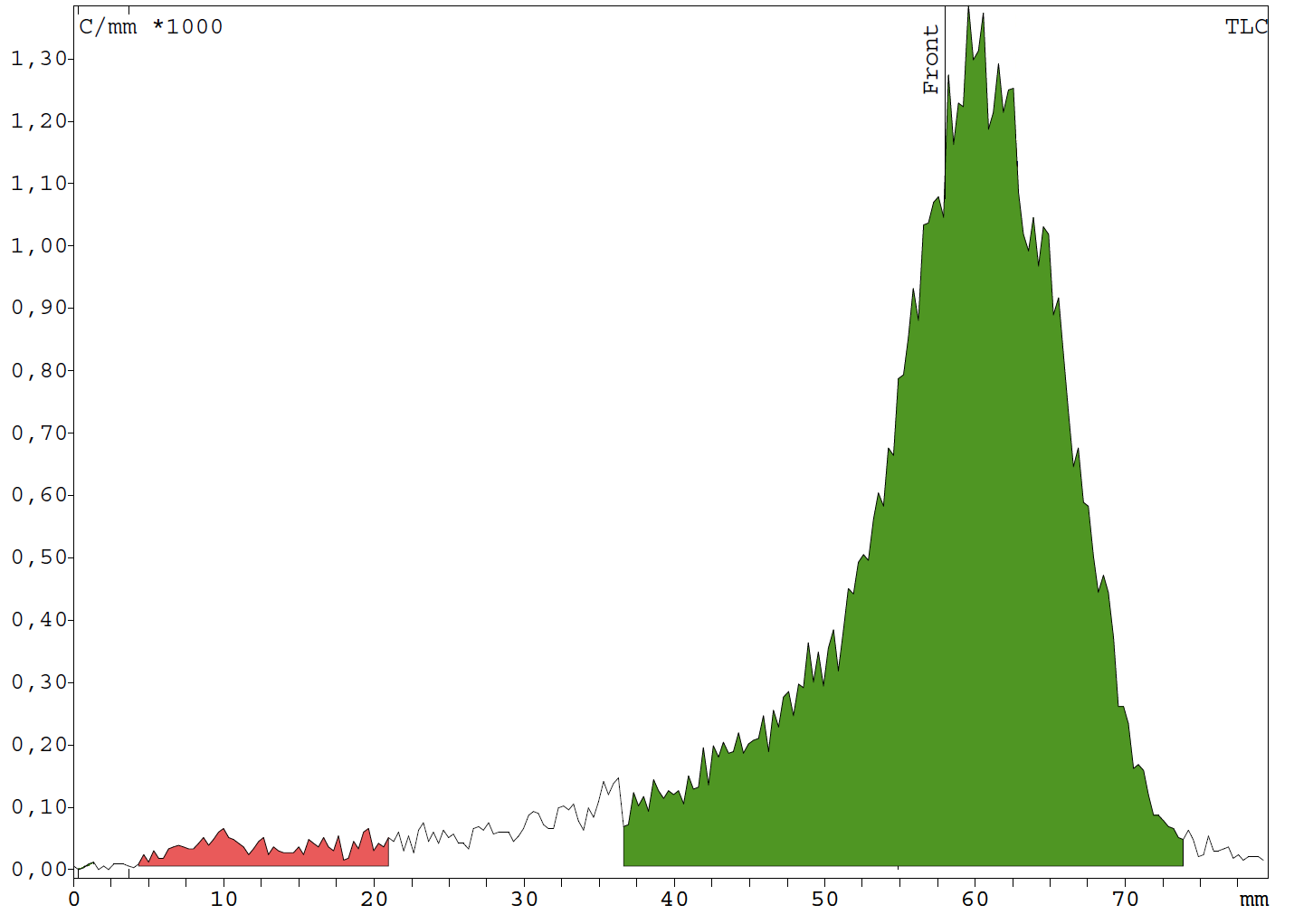

Supplement: Supplementary file 1 [file pharmaceutics-14-02695-s001.zip › Figure S7.png]

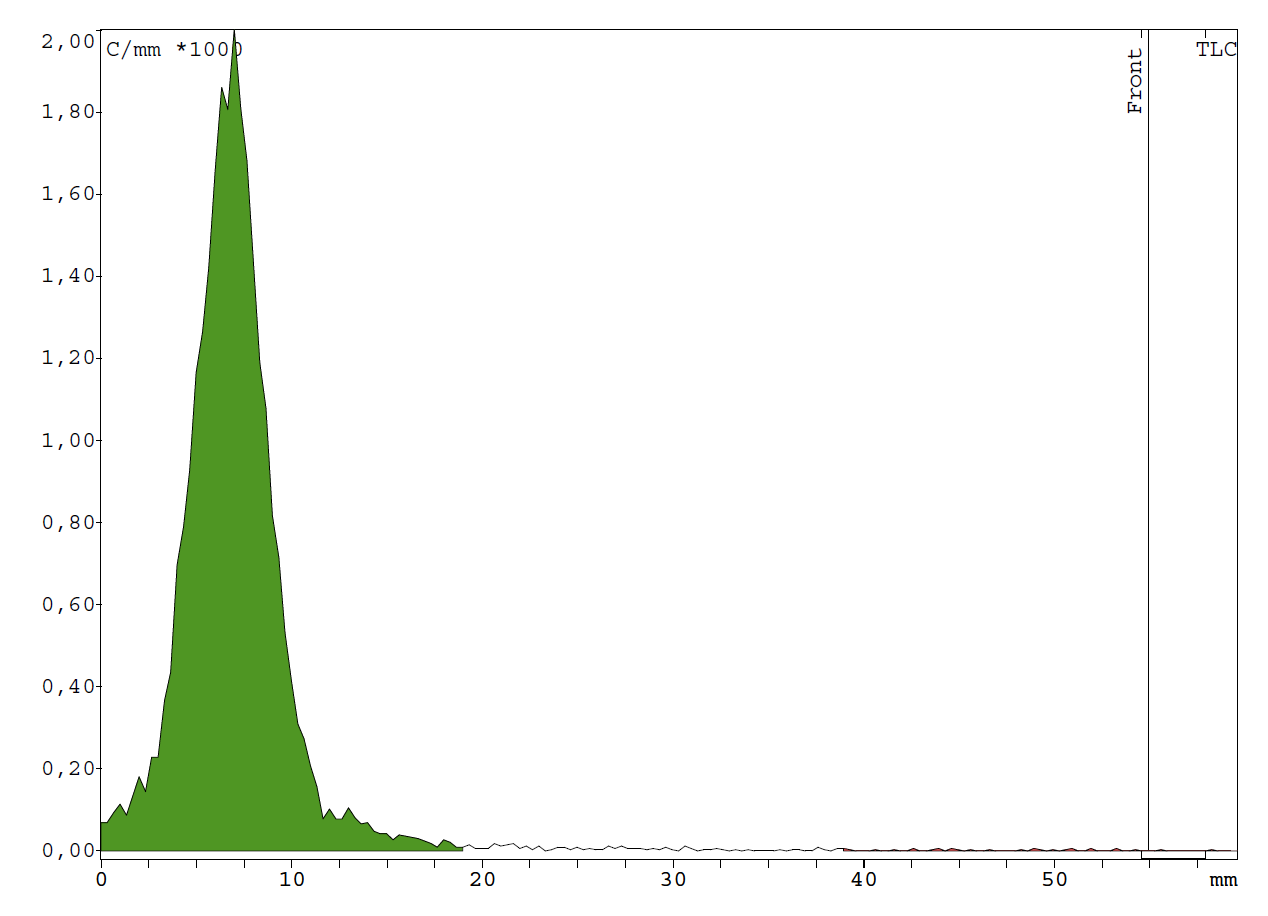

Supplement: Supplementary file 1 [file pharmaceutics-14-02695-s001.zip › Figure S8.png]

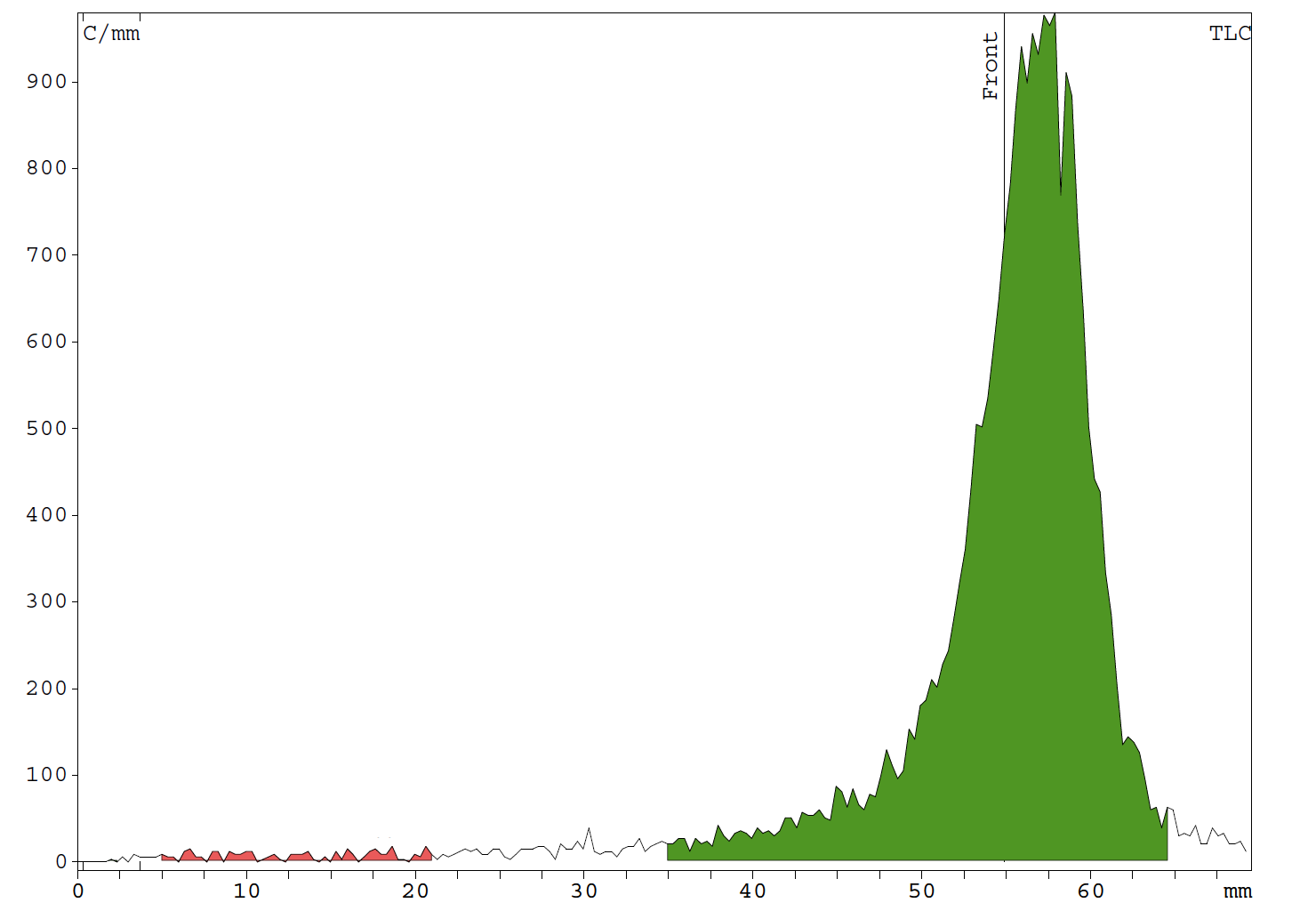

Supplement: Supplementary file 1 [file pharmaceutics-14-02695-s001.zip › Figure S9.png]
